# Characteristics and Factors Associated With Antihypertensive Medication Use in Patients Attending Peruvian Health Facilities

**DOI:** 10.7759/cureus.1011

**Published:** 2017-02-03

**Authors:** Christian R Mejia, Virgilio E Failoc-Rojas, Edison So, Carmen Cervantes, Antonio J Aspajo, Jesus Galileo Leandro, Jhomar Cordova-De La Cruz, Julio C Charri, Kevin E García-Auqui, Lelis Gabriela Coronel-Chucos, Luz Delia Justo-Pinto, Marisol Stefanie Mamani-Apaza, Neil Arón Paz-Campos, Ricardo Correa

**Affiliations:** 1 Escuela de Medicina Humana, Universidad Continental, Huancayo, Peru; 2 Medicine, Universidad Nacional Pedro Ruiz Gallo. Lambayeque, Peru; 3 Endocrinology and Metabolism, The Warren Alpert Medical School of Brown University; 4 Medicine, Aventura Hospital; 5 Medicine, Universidad Nacional de la Amazonía Peruana. Loreto, Peru.; 6 Medicine, Universidad Nacional Daniel Alcides Carrión. Cerro de Pasco, Peru.; 7 Medicine, Sociedad Científica de Estudiantes de Medicina del Centro, Universidad Nacional del Centro del Perú. Huancayo, Junín, Perú.; 8 Medicine, Escuela Académico Profesional de Medicina Humana, Universidad Continental. Huancayo, Junín, Perú.; 9 Medicine, Facultad de medicina, Universidad Nacional del Altiplano, Puno, Peru; 10 Facultad de Medicina Humana, Universidad Peruana Los Andes; 11 NICHD, National Institute of Health

**Keywords:** hypertension, pharmacologic treatment, multicenter, peru

## Abstract

**Introduction:**

Hypertension is a very common disease worldwide, and medication is needed to prevent its short-term and long-term complications. Our objective was to determine the characteristics and factors associated with antihypertensive medication use in patients attending Peruvian health facilities.

**Materials & Methods:**

We performed a multicenter, cross-sectional study with secondary data. We obtained self-reported antihypertensive medication from patients attending health facilities in 10 departments of Peru. We looked for associations of the antihypertensive treatment according to sociopathological factors and obtained p values using generalized linear models.

**Results:**

Of the 894 patients with hypertension, 61% (547) were women and 60% (503) were on antihypertensive treatment, of which 82% (389) had monotherapy and 52% (258) had recently taken their medication. Antihypertensive treatment was positively correlated with the patient's age (adjusted prevalence ratio [aPR]: 1.01; 95% confidence interval [CI]: 1.007 to 1.017; p value < 0.001), diabetes (aPR: 1.31; 95% CI: 1.11 to 1.55; p value = 0.001) and cardiovascular disease (aPR: 1.38; 95% CI: 1.26 to 1.51; p value < 0.001). Conversely, the frequency of antihypertensive treatment decreases with physical activity (aPR: 0.80; 95% CI: 0.70 to 0.92; p value = 0.001).

**Conclusion:**

Patients who have comorbidities and advanced age are more likely to be on antihypertensive treatment. In contrast, patients with increased physical activity have a lower frequency of antihypertensive treatment. It is important to consider these factors for future preventive programs and to improve therapeutic compliance.

## Introduction

The complications of high blood pressure, if not addressed, promptly become the leading cause of death worldwide [1-2]. Treatment ranges from lifestyle modifications [3] to pharmacologic therapy [4]. Specific blood pressure targets should be achieved according to age and comorbidities [5] because the excessive reduction is also associated with increased morbidity and mortality [6]. This represents a current large-scale medical challenge. The inadequate control of high blood pressure worldwide has led to increased cardiovascular events [7-8] and/or damage to target organs [9]. Almost half of hypertensive patients are not on treatments, and a higher proportion of patients with hypertension, diabetes, and kidney disease are not on treatment. Therefore, a simple treatment is recommended to improve compliance [10] and early initiation of medication for higher risk patients [11].

Most studies on this subject, especially in our environment, address factors associated with hypertension and its consequences [5,12]. Identifying factors associated with the use of antihypertensive medications is necessary for professionals and institutions involved to manage this population properly [13]. Therefore, multinational institutions have established objectives that promote research focused on the prevention and control of noncommunicable diseases [14]. Hence, the aim of this study is to determine the characteristics and factors associated with antihypertensive medication use in patients attending Peruvian health care facilities.

## Materials and methods

### Study design

An analytical transversal study derived from secondary data was performed. The main objective was to determine the characteristics and factors associated with antihypertensive medication use in patients attending Peruvian health facilities.

### Population and sample

Data were obtained from patient surveys in health facilities in the following cities of Peru: Piura, Chiclayo, Lima, Huancayo, Iquitos, Cajamarca, Huanuco, Cusco, Puno, Cerro de Pasco, and Rinconada-Puno. The samples were not randomized. Data from all adult patients who answered the questionnaire voluntarily were included as well as those with a prior diagnosis of hypertension (to determine the amount of and associated factors with medication). People who did not answer medication-related questions were excluded.

### Variables

The primary endpoint was receiving antihypertensive medication. This information was obtained from the main database by asking if the person had any drug to control blood pressure (response categories: YES or NO). Other variables considered to characterize medication were the number of drugs taken (by specifying all drugs that they took) and if they had taken any medication on the day of the survey.

With regard to factors associated with medication, we included: gender (male/female), age (a quantitative variable considered for the analysis), medical condition, treatment of comorbidities (diabetes mellitus, heart disease, hypercholesterolemia, and hypertriglyceridemia), and performance of physical activity or any sports (corroborated by asking type, years, and hours practiced per month). All these data were taken from the database, which was built from self-reports.

### Ethics and procedures

Primary research was accepted by the institutional review board (IRB) of the Hospital Nacional San Bartolome and Hospital Regional de Lambayeque, entities accredited by the Instituto Nacional de Salud. Informed patient consent was obtained. After receiving the database, quality control was performed separately by two authors, who compared the database data with the survey data and merged it into one database (for a subsequent review if data matched). All this was done in an Excel 2010 data sheet (Microsoft, Redmond, Washington, USA). Following this, we performed statistical analysis.

### Analysis of data

The data were processed statistically in Stata software, version 11.1 (StataCorp, College Station, Texas, USA). For the description of the numerical variable age, its normality was determined with the Shapiro-Wilk statistical test, which allowed the establishment of median and ranges. For the description of categorical variables, frequencies and percentages were used.

For bivariate analysis, we obtained p values, crude prevalence ratios (cPRs), aPRs, and 95% CIs. Generalized linear models were used with Poisson distribution and log link function. Also, we considered the uniqueness of the surveyed populations, so we used the headquarters of the respondent as a cluster. To avoid nonconvergence in the multivariate analysis, we included medication for each comorbidity and physical activity in the final model (independent from medical conditions or sport). Confidence intervals of 95% and p values <0.05 were recognized as statistically significant.

## Results

Out of the 894 patients with hypertension, 61% (547) were women, and the median age was 57 years. Sixty percent (503) of patients were recorded to be receiving antihypertensive treatment; among this group, 82% (389) were on monotherapy, and 52% (258) had recently taken their medication (Table 1).

**Table 1:** Characteristics of the respondents and the antihypertensive treatment they received *Median and range HTN = hypertension; ARA-II = angiotensin II receptor antagonist; ACE = angiotensin converting enzyme

Variable	N	%
Gender		
Female	547	61.2
Male	347	38.8
Age (years)*	57	18-93
Receives anti-HTN treatment		
Yes	503	60.5
No	329	39.5
Number of anti-HTN drugs		
1	414	82.8
2	75	15
3	8	1.6
4	3	0.6
Had anti-HTN treatment today		
Yes	258	51.7
No	241	48.3
Type of drug		
ARA-II	93	18.6
Beta blocker	15	3
Calcium antagonist	23	4.6
ACE inhibitor	288	57.6
Thiazide​	10	2
ARA-II + beta blocker	3	0.6
ARA-II + calcium antagonist	15	3
ARA-II + thiazide	5	1
ARA-II + ACE inhibitor	8	1.6
ACE + beta blocker	3	0.6
ACE + calcium antagonist	8	1.6
ACE + thiazide	18	3.6
Triple therapy, quadruple therapy	8, 3	1.6, 0.6

**Figure 1:**
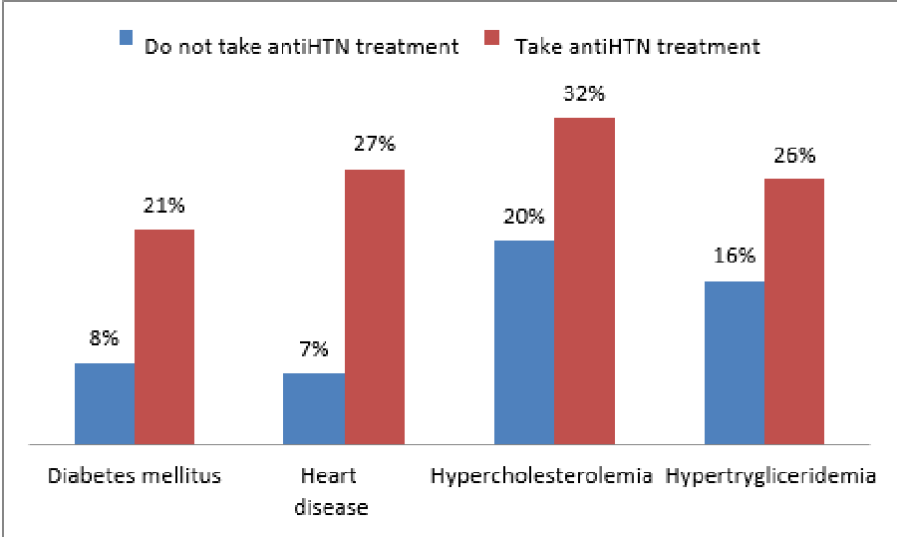
Percentage of people taking antihypertensive treatment related to comorbidities

Figure 1 shows the percentage of people taking antihypertensive treatment and having comorbidities.

Antihypertensive treatment was positively correlated to the patient's age (p < 0.001) and known diagnosis of the following medical conditions and treatment timing: diabetes mellitus (p = 0.001 and < 0.001, respectively), heart disease (p < 0.001 and < 0.001, respectively), hypercholesterolemia (p = 0.017 and < 0.001, respectively), and hypertriglyceridemia (p = 0.043 and < 0.001, respectively). Moreover, frequency of antihypertensive medication use decreased when performing physical activity (p < 0.001) and any sports (p = 0.004). See Table 2.

**Table 2:** Bivariate analysis of factors associated with receiving antihypertensive treatment Confidence interval (95% CI) and p value obtained from generalized linear models with Poisson distribution, log link function, and headquarters of the respondent as clusters. HTN = hypertension; cPR = crude prevalence ratio *Mean and standard deviation

Variable	Patients with HTN N (%)				cPR (95% CI)		*p v*alue	
	With treatment	Without treatment						
Gender								
Female	306 (60.8)		202 (61.4)		0.99 (0.77-1.28)		0.943	
Male	197 (39.2)		127 (38.6)					
Age (years)*	60.2 (15.0)		50.4 (16.4)		1.02 (1.01-1.03)		<0.001	
Diabetes mellitus								
Diagnosed	106 (21.1)		26 (7.9)		1.42 (1.23-1.63)		0.001	
Receives treatment	94 (18.7)		16 (4.9)		1.51 (1.25-1.82)		<0.001	
Heart disease								
Diagnosed	138 (27.4)		22 (6.7)		1.59 (1.40-1.80)		<0.001	
Receives treatment	112 (22.3)		11 (3.3)		1.65 (1.45-1.88)		<0.001	
Hypercholesterolemia								
Diagnosed	160 (31.8)		67 (20.4)		1.24 (1.04-1.49)		0.017	
Receives treatment	105 (20.9)		30 (9.1)		1.36 (1.23-1.52)		<0.001	
Hypertriglyceridemia								
Diagnosed	130 (25.9)		53 (16.1)		1.24 (1.01-1.52)		0.043	
Receives treatment	86 (17.2)		22 (6.7)		1.39 (1.25-1.53)		<0.001	
Other disease								
Chronic	231 (48.3)		147 (46.8)		1.02 (0.88-1.20)		0.764	
Cardiovascular	43 (9.0)		20 (6.4)		1.14 (0.94-1.39)		0.181	
Performs								
Physical activity	137 (27.2)		155 (47.1)		0.69 (0.58-0.82)		<0.001	
Any sport	72 (14.3)		89 (27.1)		0.70 (0.54-0.89)		0.004	

When performing the multivariate model, increased age in years was associated with higher frequency of medication use (aPR: 1.01; 95% CI: 1.007 to 1.017; p value < 0.001). Similarly, antihypertensive medication use was more frequent in patients who were receiving treatment for diabetes (aPR: 1 31; 95% CI: 1.11 to 1.552; p value = 0.001) and in those with heart disease (aPR: 1.38; 95% CI: 1.26 to 1.51; p value < 0.001). However, those doing physical activity took less medication (aPR: 0.80; 95% CI: 0.70 to 0.92; p value = 0.001), adjusted for receiving treatment for hypercholesterolemia or hypertriglyceridemia (Table 3).

**Table 3:** Multivariate analysis of factors associated with receiving antihypertensive treatment Confidence interval (95% CI) and p value obtained from generalized linear models with Poisson distribution, log link function, and headquarters of the respondent as clusters. aPR = adjusted prevalence ratio *Mean and standard deviation

Variable	aPR (95% CI)	p value
Age (years)*	1.01 (1.007-1.017)	<0.001
Receives treatment for:		
Diabetes mellitus	1.31 (1.11-1.55)	0.001
Heart disease	1.38 (1.26-1.51)	<0.001
Hypercholesterolemia	1.08 (0.90-1.29)	0.398
Hypertriglyceridemia	1.16 (0.98-1.37)	0.075
Performs physical activity	0.80 (0.70-0.92)	0.001

## Discussion

Hypertension represents a worldwide epidemic, with a global prevalence of 40% [15]. Hypertension can cause direct and indirect complications, including cardiovascular disease, and accounts for 9.4 million annual deaths [15-19]. Based on these facts, it is of utmost importance to treat hypertension to reduce or prevent serious complications [20].

In our study, only 60% of hypertensive patients were receiving treatment. This is consistent with other studies that show hypertensive patients have poor adherence to treatment [17-19], which may be the primary cause of uncontrolled hypertension [16]. On the other hand, there may be multiple reasons for nonadherence to medications such as poor access to healthcare, cost of medicine, etc. These data can help physicians develop strategies to improve medication adherence by identifying the profile of patients who do not comply with treatment [20] to generate intervention policies and improve care in this patient population. A meta-analysis of 22 studies by Nielsen et al. showed that social and economic factors are the most reported factors of nonadherence in low- and middle-income countries, followed by condition-related factors (e.g., comorbidities and medication side effects) and patient-related factors (e.g., knowledge and attitude) [15]. 

Our study showed that patients with increased age are more likely to be on antihypertensive medication. Moreover, hypertensive patients have a higher rate of comorbidities, which makes it more difficult to address and avoid complications [21-22]. Gender showed no influence on the pharmacological control of hypertension in our study, which conflicts with reports from many other studies [22-23] that display less compliance in males. However, there are other studies that align with our gender findings [21]. Therefore, we recommend further study of the association between these two variables in the pharmacological management of hypertensive patients.

Our study also revealed that carrying a diagnosis and receiving treatment for diabetes mellitus or heart disease increases the frequency of antihypertensive medication use. This is consistent with studies showing that patients tend to take their medications to avoid similar complications [24], especially if they have concurrent heart diseases, to avoid cardiovascular complications [25].

Finally, this study showed that physical activity was associated with a lower frequency of antihypertensive medication use. This may be because patients with constant physical activity tend to improve their blood pressure, as shown in some revisions of the subject [26]. This is further supported by a meta-analysis of randomized controlled trials showing that aerobic exercise of at least four weeks significantly reduced resting and ambulatory blood pressure [27-28].

Our study has a selection bias because the sample was not randomized, so confidence intervals are used merely for comparison with other studies and estimation of the results obtained by the amplitude range. Furthermore, the data we collected is mainly based on patient surveys, which may be less accurate than patient records. On the other hand, this study has multiple strengths: it reflects data from multiple health centers in Peru; the most important variables related to the disease were considered; and it is the first report in our area that reviews the association of antihypertensive medication use with patient characteristics.

## Conclusions

Based on revised data, we conclude that hypertensive patients in Peru have a poor medication adherence. Increasing age, diabetes, and heart disease are associated with the increased use of antihypertensive medications. On the other hand, increased physical activity decreases the use of antihypertensive medications. All these factors should be considered during efforts to improve programs on patient compliance.

Further studies are needed to identify the factors affecting medication adherence in the hypertensive patients of Peruvian health facilities. This will enable us to create a strategy or solution for each of these factors, subsequently improve medication compliance, and finally, reduce the complications from hypertension.
